# Supplementary material for: Association between Adverse Events and Prognosis in Patients with Hepatocellular Carcinoma Treated with Atezolizumab Plus Bevacizumab: A Multicenter Retrospective Study
Source: Cancers (Basel). 2022 Sep 1;14(17):4284. doi: 10.3390/cancers14174284 (PMC9454839; doi:10.3390/cancers14174284)
Supplement: Supplementary file 1 [file cancers-14-04284-s001.zip › cancers-1851638-supplementary.pdf]

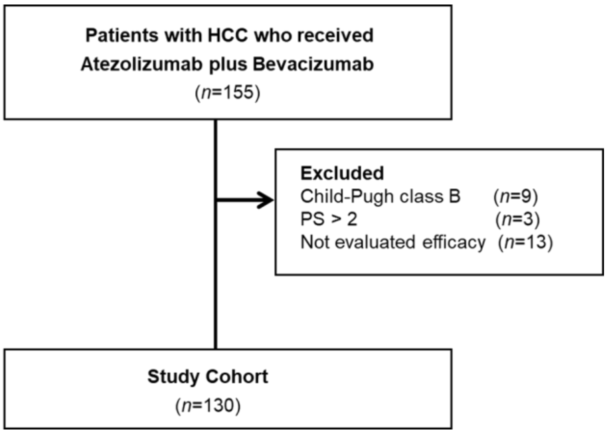

Figure S1. Study design. A total of 153 patients with HCC were enrolled. In the course of the study, 25 patients were excluded, and 130 patients with HCC were analyzed in this study. HCC, hepatocellular carcinoma.

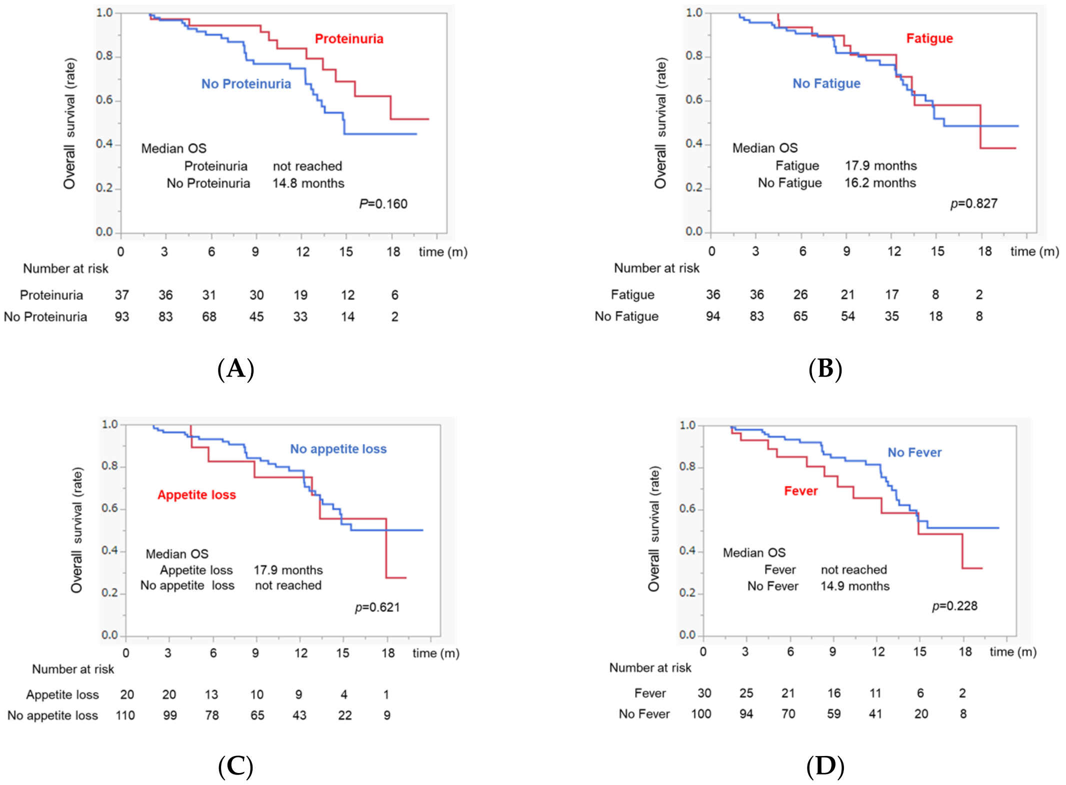

Figure S2. Overall survival time in patients who developed each AE in atezo/beva treatment. (**A**) Kaplan–Meier curves for overall survival according to with and without proteinuria. The red line indicates the proteinuria group. The blue line indicates the no proteinuria group. (**B**) Kaplan–Meier curves for overall survival according to with and without fatigue. The red line indicates the fatigue group. The blue line indicates the no fatigue group. (**C**) Kaplan–Meier curves for overall survival according to those with and without appetite loss. The red line indicates the decreased appetite group. The blue line indicates the no appetite loss group. (**D**) Kaplan–Meier curves for overall survival according to with and without fever. The red line indicates the fever group. The blue line indicates the no fever group. AE, adverse events; atezo/beva, atezolizumab plus bevacizumab.

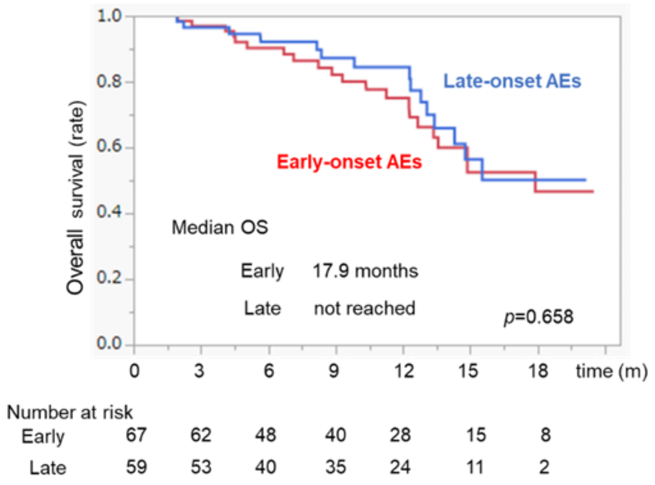

Figure S3. Overall survival time in patients with HCC according to early and late onset AEs. Kaplan–Meier curves for overall survival according to early-onset of AEs or late-onset of AEs in patients with HCC treated with atezo/beva. The blue line indicates the late-onset of AEs group. The red line indicates the early-onset of AEs group. HCC, hepatocellular carcinoma; AE, adverse events; atezo/beva, atezolizumab plus bevacizumab.

Table S1. The reasons for treatment discontinuation in atezo/beva.

**Factor**	**(n = 96)**
Progressive disease	50 (52.1%)
Adverse events	31 (32.3%)
Fatigue	8 (8.3%)
Liver injury	6 (6.2%)
Gastrointestinal bleeding	4 (4.2%)
Proteinuria	2 (2.1%)
Ascites	2 (2.1%)
Fever	2 (2.1%)
Pneumoniae	2 (2.1%)
Ascites	1 (1.1%)
Others	4 (4.2%)
Conversion	10 (10.4%)
Operation	5 (5.2%)
RFA	2 (2.1%)
TACE	3 (3.1%)
Sufficient therapeutic effect	3 (3.1%)
Others	2 (2.1%)

Abbreviations: Atezo/Beva, atezolizumab plus bevacizumab; RFA: radiofrequency ablation, TACE; transarterial chemoembolization.

Table S2. Therapeutic responses according to AEs (n = 130).

	All	ORR (n = 35)	p	DCR (n = 114)	p
Liver injury (Presence/Absence)	57/73	13/22 (22.8%/30.1%)	0.349	48/66 (84.2%/90.4%)	0.285
Hypertension (Presence/Absence)	54/76	17/18 (31.4%/23.6%)	0.323	53/61 (98.1%/80.2%)	0.002
Proteinuria (Presence/Absence)	37/93	9/26 (24.3%/27.9%)	0.673	33/81 (89.1%/87.1%)	0.743
Fatigue (Presence/Absence)	36/94	14/21 (38.8%/22.3%)	0.067	32/82 (88.8%/87.2%)	0.797
Skin disorder (Presence/Absence)	32/98	11/24 (34.4%/24.4%)	0.273	30/84 (93.7%/85.7%)	0.229
Fever (Presence/Absence)	30/100	9/26 (30.0%/26.9%)	0.664	25/89 (83.3%/89.0%)	0.407

Abbreviations: RECIST, response evaluation criteria in solid tumors; ORR, objective response rate; DCR; disease control rate.

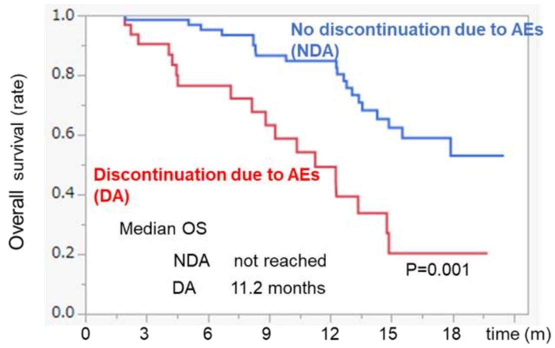

Number at risk							
NDA	65	63	57	51	39	22	8
DA	31	27	21	14	11	3	1

Figure S4. Overall survival time in patients with HCC who did or did not require discontinuation due to AEs. Kaplan–Meier curves for overall survival according to discontinuation due to AEs or no discontinuation due to AEs in patients with HCC treated with atezo/beva. The blue line indicates the no discontinuation due to AEs group. The red line indicates the discontinuation due to AEs group. HCC, hepatocellular carcinoma; AE, adverse events; atezo/beva, atezolizumab plus bevacizumab.
